# Plant Proteinase Inhibitor BbCI Modulates Lung Inflammatory Responses and Mechanic and Remodeling Alterations Induced by Elastase in Mice

**DOI:** 10.1155/2017/8287125

**Published:** 2017-03-30

**Authors:** Rafael Almeida-Reis, Osmar A. Theodoro-Junior, Bruno T. M. Oliveira, Leandro V. Oliva, Alessandra C. Toledo-Arruda, Camila R. Bonturi, Marlon V. Brito, Fernanda D. T. Q. S. Lopes, Carla M. Prado, Ariana C. Florencio, Mílton A. Martins, Caroline A. Owen, Edna A. Leick, Maria L. V. Oliva, Iolanda F. L. C. Tibério

**Affiliations:** ^1^Department of Medicine, School of Medicine, University of São Paulo, São Paulo, SP, Brazil; ^2^Division of Pulmonary and Critical Care Medicine, Brigham and Women's Hospital, Harvard Medical School, Boston, MA, USA; ^3^Department of Biochemistry, Federal University of São Paulo, São Paulo, SP, Brazil; ^4^Department of Biological Sciences, Federal University of São Paulo, São Paulo, SP, Brazil; ^5^The Lovelace Respiratory Research Institute, Albuquerque, NM, USA

## Abstract

*Background.* Proteinases play a key role in emphysema.* Bauhinia bauhinioides cruzipain* inhibitor (BbCI) is a serine-cysteine proteinase inhibitor. We evaluated BbCI treatment in elastase-induced pulmonary alterations.* Methods.  C57BL*/*6* mice received intratracheal elastase (ELA group) or saline (SAL group). One group of mice was treated with BbCI (days 1, 15, and 21 after elastase instillation, ELABC group). Controls received saline and BbCI (SALBC group). After 28 days, we evaluated respiratory mechanics, exhaled nitric oxide, and bronchoalveolar lavage fluid. In lung tissue we measured airspace enlargement, quantified neutrophils, TNF*α*-, MMP-9-, MMP-12-, TIMP-1-, iNOS-, and eNOS-positive cells, 8-iso-PGF2*α*, collagen, and elastic fibers in alveolar septa and airways. MUC-5-positive cells were quantified only in airways.* Results.* BbCI reduced elastase-induced changes in pulmonary mechanics, airspace enlargement and elastase-induced increases in total cells, and neutrophils in BALF. BbCI reduced macrophages and neutrophils positive cells in alveolar septa and neutrophils and TNF*α*-positive cells in airways. BbCI attenuated elastic and collagen fibers, MMP-9- and MMP-12-positive cells, and isoprostane and iNOS-positive cells in alveolar septa and airways. BbCI reduced MUC5ac-positive cells in airways.* Conclusions.* BbCI improved lung mechanics and reduced lung inflammation and airspace enlargement and increased oxidative stress levels induced by elastase. BbCI may have therapeutic potential in chronic obstructive pulmonary disease.

## 1. Introduction

Chronic obstructive pulmonary disease (COPD) is a major cause of chronic respiratory morbidity and mortality and is the fifth leading cause of death worldwide [1]. A proteinase-antiproteinase imbalance is the most widely accepted hypothesis to explain lung tissue destruction. In this context, elastase secreted by neutrophils and macrophages may play an important role in lung tissue destruction [2, 3].

Current COPD management involves patients reducing their exposure to cigarette smoke, using inhaled bronchodilators and corticosteroid, and receiving prompt treatment of acute exacerbations [4]. However, we currently lack treatments that reduce the progression or adequately suppress the inflammation present in the small airways and lung parenchyma of COPD patients. To better understand the pathogenesis of emphysema, the elastase-induced model was developed 50 years ago. It is a simple and widely used method to induce emphysema in animals. This model has also been used to test potential new therapeutic agents for COPD [5].

Proteinase inhibitors have been considered potential treatments that could be used to modify the course of COPD. In addition to degrading extracellular matrix proteins, proteinases have important signaling functions in cell death, cell proliferation, DNA replication, inflammatory response, and tissue remodeling [6]. Thus, by inhibiting proteolytic enzymes implicated in COPD pathogenesis, proteinase inhibitors could reduce the progression of disease [7]. Some proteinase inhibitors are also found in plants. Their role in preventing excessive proteolysis during tissue inflammation has been recently investigated [8, 9].

*Bauhinia* is a plant genus from subfamily Caesalpinioideae, which comprises more than 600 species found in tropical and subtropical forests. Many proteinase inhibitors have been isolated from this genus, particularly from* Bauhinia bauhinioides*.* Bauhinia bauhinioides cruzipain* inhibitor (BbCI) is an 18 kDa Kunitz-type proteinase inhibitor isolated from* Bauhinia bauhinioides* seeds [9]. BbCI inhibits the activity of different serine proteinases, such as human neutrophil elastase, porcine pancreatic elastase, and cathepsin G. BbCI also inhibits the activity of cysteine proteinases, such as cathepsin L, cruzipain, and cruzain [10]. The goal of this study was to test the hypothesis that* Bauhinia bauhinioides cruzipain* inhibitor (BbCI) limits elastase-induced alterations in pulmonary mechanics, emphysema development, lung inflammation, extracellular matrix remodeling, and oxidative stress.

## 2. Materials and Methods

### 2.1. Animals and Study Design

Male C57Bl/6 mice (20–25 g) were maintained in an animal facility with a 12-hour light-dark cycle and fed with water and chow* ad libitum*. Animals received human care in compliance with the “Guide for Care and Use of Laboratory Animals” (National Institutes of Health, publication 86-23, revised 1985). Protocols were approved by the Ethical Committee of the University of São Paulo. Animals were divided into four groups of 8 animals each: (a) animals that received a tracheal instillation and intraperitoneal injection of vehicle (SAL, *n* = 8); (b) animals that received a tracheal instillation of elastase and intraperitoneal injection of vehicle (ELA, *n* = 8); (c) animals that received a tracheal instillation of saline and intraperitoneal injection of BbCI (SALBC, *n* = 8); (d) animals that received a tracheal instillation of elastase and intraperitoneal injection of BbCI (ELABC, *n* = 8).

### 2.2. Elastase-Induced Emphysema Mouse Model

Six-week-old C57Bl/6 mice were anesthetized with an intramuscular injection of ketamine (40 mg/kg) and xylazine (5 mg/kg). The trachea was exposed, and each animal received porcine pancreatic elastase (0.667 UI diluted in 50 *µ*L of sterile saline; elastase type I/E-1250, Sigma-Aldrich, Saint Louis, USA) directly into trachea. After instillation, we performed a chest massage to spread the elastase in the lung. Control animals received 50 *µ*L of sterile saline using the same method.

### 2.3. Inhibitor Expression and Purification

**Figure 1:**
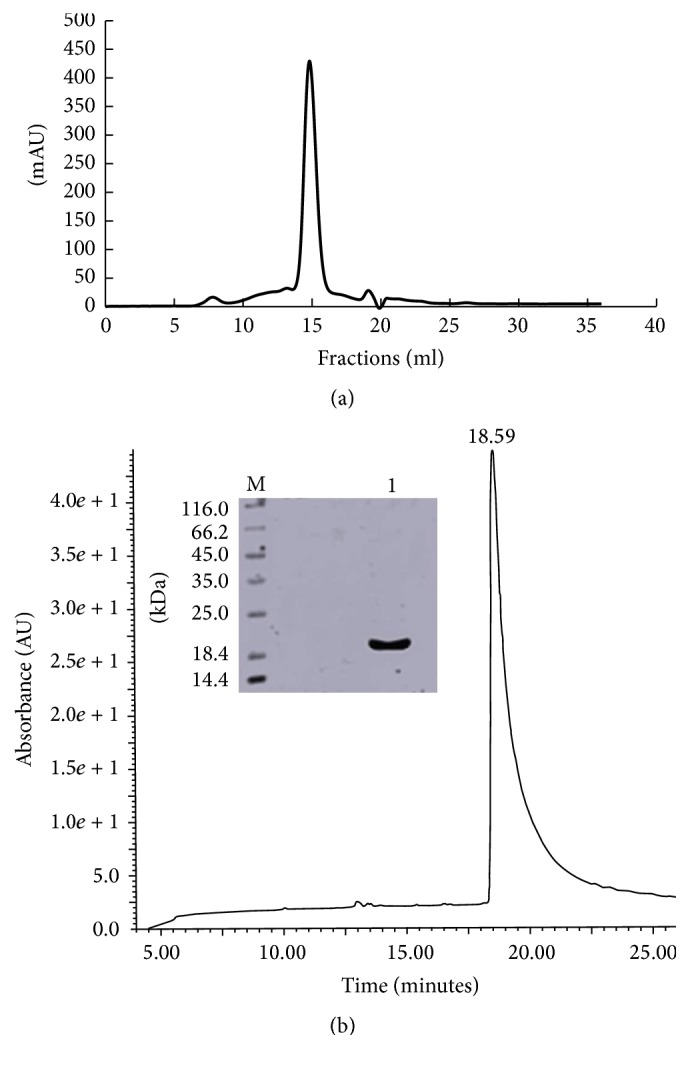
rBbCI purification from cells lysate. (a) Molecular exclusion chromatography on a Superdex G-75 10/30 column equilibrated with 50 M Tris-HCl buffer containing 150 M NaCl, pH 8.0. (b) Reverse phase chromatograph, HPLC system *μ*-BondapakC18. SDS-PAGE (15%) from reverse phase chromatography on C18. (M) Molecular mass standards; (1) rBbCI (30 *μ*g) treated with *β*-mercaptoethanol.

Overexpression of the rBbCI in* E. coli* BL21 (DE3) cells and its purification were carried out according to Ulian Araújo et al. [11]. Briefly, cells containing the target gene cloned into the expression vector pET28a (Novagen) were grown in Luria-Bertani medium supplemented with 30 *µ*g/mL kanamycin (Invitrogen) at 37°C to an optical density OD600 nm of 0.4, followed by induction of the fusion-protein expression with isopropyl*β*-D-thiogalactopyranoside (Invitrogen). IPTG was added at a final concentration of 1 M and the culture was grown for additional 3 hours and centrifuged (4000 ×g, 20 min, 4°C) (Hitachi Himac CR21-GIII). The pellets were resuspended in 10 mL of 0.05 M Tris/HCl, pH 8.0 buffer, and 0.15 M NaCl. Bacterial cells disruption by sonication (Unique, Campinas, Brazil) were lysed in 0.5% Triton X-114 (Sigma-Aldrich) (ten cycles of ultrasound, 30 s, 30 W) and centrifuged (4000 ×g, 20 min, 4°C). LPS was removed by addition of chloroform (3 : 1, v/v) at 4°C after centrifugation. The protein was further purified by Ni–NTA affinity chromatography and eluted with an imidazole gradient (100–250 M) in the same sonication buffer. The imidazole was removed by being dialyzed against 0.05 M Tris/HCl, pH 8.0, and 0.15 M NaCl buffer. The His-tag was removed by digestion with 0.5 U thrombin (GE Healthcare) per milligram of fusion protein for 4 h at 18°C. The final step of purification involved molecular exclusion chromatography on a Superdex 75 HR 10/30 column (AKTA Purifier, GE Healthcare) in 0.05 M Tris/HCl, pH 8.0, and 0.15 M NaCl to separate the rBbCI protein from His-tag; the homogeneity of the preparation was analyzed by SDS-PAGE and on *μ*-Bondapak C18 reverse phase column (Figure 1).

### 2.4. Treatment with BbCI

**Figure 2:**
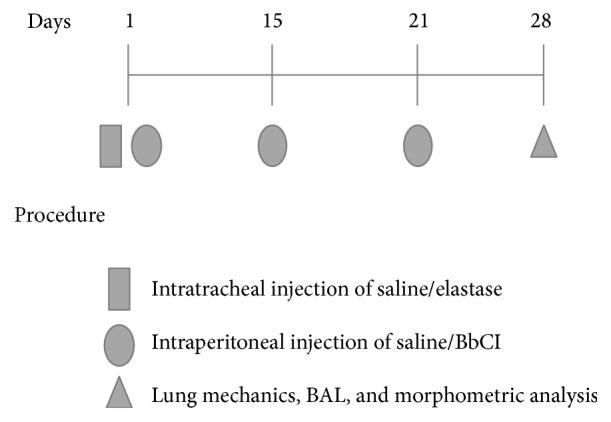
Timeline of the experimental protocol. On the first day of protocol, animals received intratracheal instillation of elastase or vehicle. Two hours after the intranasal instillation, animals received an intraperitoneal injection of treatment [BbCI (SALBC and ELABC) or saline (ELA and SAL)]. On the fifteenth day, animals received the second dose of treatment [BbCI (SALBC and ELABC) or saline (ELA and SAL)]. On the twenty-first day, animals received the last dose of BbCI [BbCI (SALBC and ELABC) or saline (ELA and SAL)]. On the twenty-eighth day the animals were anesthetized and tracheostomized and the evaluation of the lung mechanics and the BAL was performed; the lungs were removed to do the morphometric analysis.

Two hours after the elastase instillation, animals received the first dose of recombinant the* Bauhinia bauhinioides cruzipain* inhibitor. On day 15 animals received the second dose and on day 21 they received the third dose. Each animal received 2 mg/kg of BbCI diluted in 50 mL of vehicle (saline) for each dose. In totality each animal received 6 mg/kg of* BbCI*. This dose was used in other studies evaluating BbCI treatment [12]. Because the effect of BbCI is dose-dependent, the animals received three doses (Figure 2).

### 2.5. Respiratory Mechanics Evaluation

Twenty-eight days after the elastase or saline instillation, mice were anesthetized with pentobarbital sodium (50 mg/kg, ip) and tracheostomized. Lung mechanics were measured using a computer-controlled small animal ventilator (FlexiVent, Scireq, Montreal, Canada). Animals were ventilated at 60 breaths/min with a tidal volume of 20 mL·kg^−1^. Oscillatory lung mechanics were performed by producing flow oscillations at different prime frequencies (from 0.25 to 19.625 Hz). Pressure and flow data were obtained. Airway impedance was calculated at each frequency [13]. Airway resistance (Raw), tissue elastance (Htis), tissue damping (Gtis), dynamic resistance (Rrs), and dynamic elastance (Ers) parameters were obtained by using a constant phase model that can separately analyze airways and tissue. After these procedures, mice were killed by exsanguination.

### 2.6. Bronchoalveolar Lavage

A BAL was performed by injecting 0.5 mL of sterile saline into the lungs through the tracheal cannula and withdrawing the fluid into a tube on ice. The recovered volume was approximately 90% of the volume injected. This procedure was repeated three times. The recovered fluid was centrifuged at 800 ×g for 5 min at 5°C. The cell pellet was resuspended in 1.0 mL of physiological saline. A total cell analysis was performed using a* Neubauer* hemocytometer chamber and optical microscope with a 1000x magnification. Cell differentiation was performed using a cytocentrifuge. Slides were centrifuged at 900 ×g for 5 min and stained with Diff* Quick-Stain* reagent. A differential cell count was performed by evaluating >300 cells with an optical microscope [14].

### 2.7. Lung Histology and Immunohistochemistry

Lungs were removed and fixed at a constant pressure 20 cmH_2_O for 24 hours in 10% formaldehyde. They were then embedded in paraffin. Sections were processed, and 3–5 *μ*m sections were obtained. They were stained with hematoxylin-eosin to measure the mean linear intercept (Lm), Picrosirius to detect collagen fibers, or Weigert's Resorcin-Fuchsin to stain elastic fibers. Slides were coded and were evaluated by researchers blinded to the protocol design.

Immunohistochemistry was performed using the following antibodies: anti-Mac-2 (Cedarlene Lab, Ontario, Canada; 1 : 60000), anti-neutrophils (AbDserotec, Kidlington, UK; 1 : 400), anti-MMP-9 (Santa Cruz Biotechnology, California, USA; 1 : 500), anti-MMP-12 (Santa Cruz Biotechnology, Dallas, USA; 1 : 100), anti-TIMP-1 (LabVision NeoMarkers, Fremont, USA; 1 : 400), anti-iNOS (LabVision NeoMarkers, Fremont, USA; 1 : 500), anti-eNOS (LabVision NeoMarkers, Fremont, USA; 1 : 100), anti-TNF*α* (Santa Cruz Biotechnology, Dallas, USA; 1 : 900), anti 8-epi-PGF2*α* (Oxford Biomedical Research, Oxford, UK; 1 : 10000) and anti-MUC5ac (LabVision NeoMarkers, Fremont, USA; 1 : 400).

Immunohistochemical staining was performed using the biotin-streptavidin peroxidase method. An ABC vectastain kit (Vector Elite PK-6105, Burlingame, USA) was used as a secondary antibody. DAB (Sigma-Aldrich, USA) was used as a chromogen. Sections were counterstained with* Harris* hematoxylin (Merck, Darmstadt, Germany).

### 2.8. Morphometric Analysis

Using a conventional morphometric method with a 100 points/50 intercepts grid with a known area (10^4^ *μ*m^2^ of total area) attached to a microscope eyepiece. The number of positive cells in the alveolar septum was determined by the number of positive cells in each field divided by the number contacting the alveolar septum. The number of positive cells in the airway wall was determined by the number of positive cells in each field divided by the number contacting the airway wall area. The results were expressed as cells/area (10^4^ *μ*m^2^). Volume fraction of 8-iso-PGF2*α* and collagen and elastic fibers in the alveolar septum or airway walls was determined by dividing the number of points hitting the positive tissue by the total number of points hitting alveolar septum or airway walls. The results were expressed as percentages. A total of 10 fields for the alveolar septum and 9 fields for the airways per animal were randomly examined at a magnification of 1000x [15].

### 2.9. Data Analysis

Statistical analyses were performed using* SigmaStat Software* (SPSS Inc., Chicago, IL, USA). Normality was assessed by using a Kolmogorov-Smirnov test. Data were evaluated using a* One-Way Analysis of Variance*. Multiple comparisons were made using a* Holm-Sidak* method. Values were expressed as a mean ± standard error (SE). A *p* value < 0.05 was considered significant.

## 3. Results

### 3.1. Lung Mechanics

**Figure 3:**
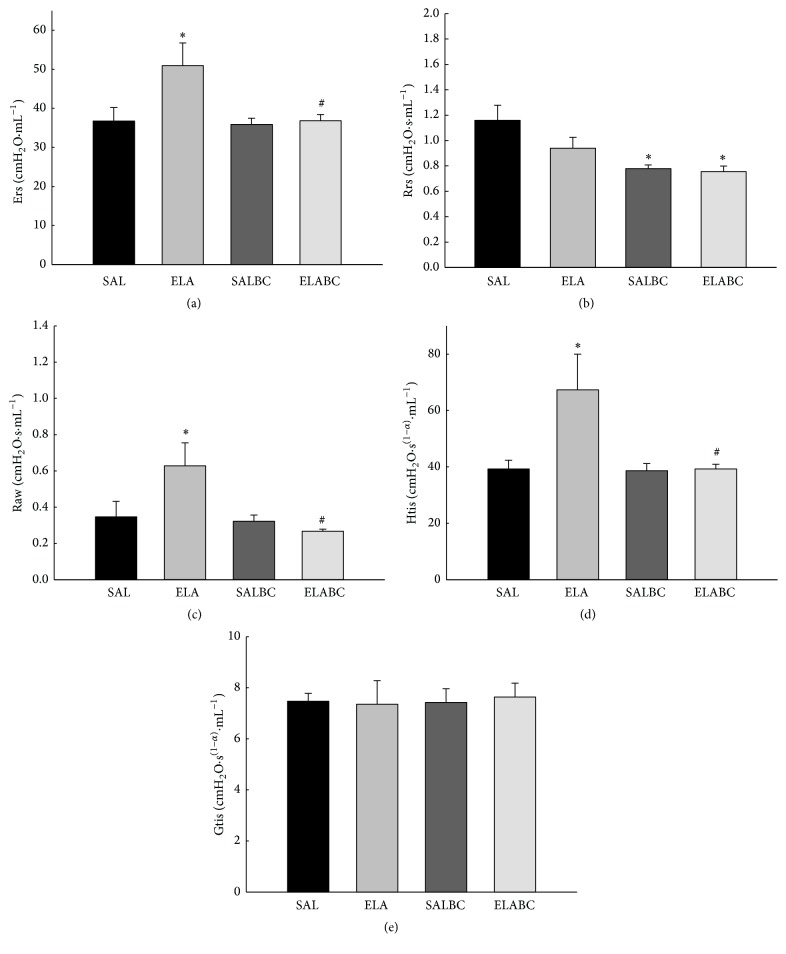
(a) Mean and SE of respiratory system elastance (Ers) of all experimental groups. ELA group was greater than control groups (^*∗*^*p* < 0.05), and ELABC had a reduction compared to ELA (^#^*p* < 0.05). (b) Mean and SE of respiratory system resistance (Rrs) in all animals. Animals that received BbCI (SALBC and ELABC) had a decrease in comparison to SAL (^*∗*^*p* < 0.05). (c) Mean and SE of airway resistance (Raw) of four experimental groups. Animals that received elastase (ELA) had greater values than SAL and SALBC groups (^*∗*^*p* < 0.05). The treatment with proteinase inhibitor (ELABC) attenuated this increase (^#^*p* < 0.05). (d) Mean and SE of tissue elastance (Htis) of all animals. Animals that received elastase (ELA) had greater values than SAL and SALBC groups (^*∗*^*p* < 0.05). The treatment with BbCI (ELABC) attenuated this increase (^#^*p* < 0.05). (e) Mean and SE of lung tissue damping (Gtis) of all experimental groups. There were no significant differences among groups.

Figure 3(a) shows the respiratory system elastance (Ers) values for all the experimental groups. There was an increase in the ELA group compared to the control groups (*p* < 0.05). The animals that received elastase and were treated with BbCI had a significant reduction in Ers compared to the ELA group (*p* < 0.05).

Figure 3(b) shows the respiratory system resistance (Rrs) values for all the animals. The animals that received BbCI (SALBC and ELABC) had decreased Rrs values compared to the SAL group (*p* < 0.05).

Figure 3(c) shows the airway resistance (Raw) values for the four experimental groups. The ELA group had increased Raw values compared to the control groups (*p* < 0.05). The animals treated with the proteinase inhibitor BbCI had decreased Raw values compared to the ELA group (*p* < 0.05) and had similar values to the controls.

Figure 3(d) shows the lung tissue elastance (Htis) values for all the experimental groups. The ELA group had increased Htis compared to the control groups (*p* < 0.05). The animals that received elastase and were treated with BbCI had similar values to the controls and had a significant attenuation compared to ELA animals (*p* < 0.05).

Figure 3(e) shows the lung tissue damping (Gtis) values for all the animals. There were no significant differences between the groups.

### 3.2. Bronchoalveolar Lavage (BAL)

**Figure 4:**
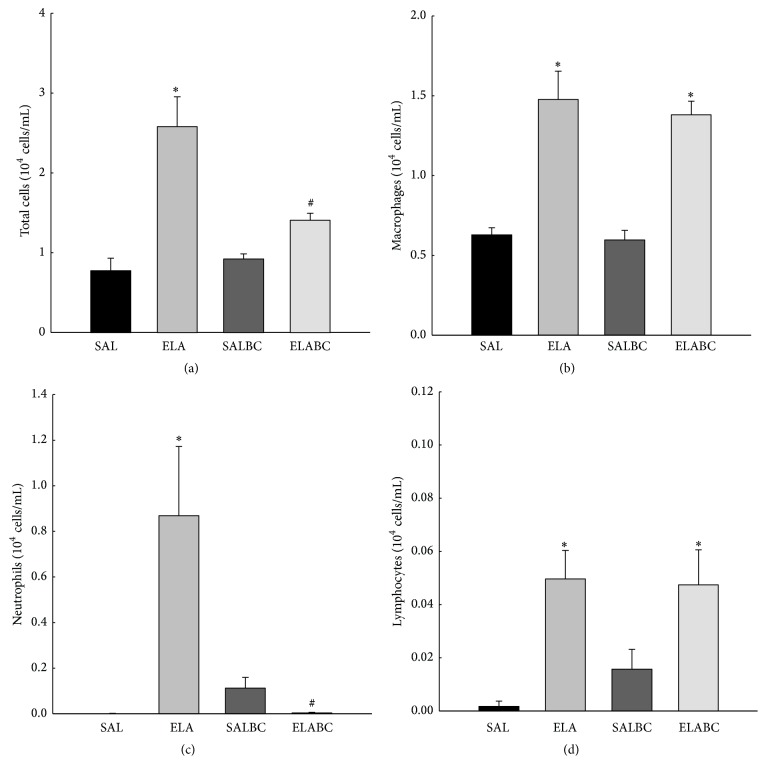
(a) Mean and SE of BAL total cells of all experimental groups. The elastase-induced group had a greater total number of cells than the control groups (^*∗*^*p* < 0.05). The treatment with BbCI (ELABC) attenuated this increase compared with ELA group (^#^*p* < 0.05). (b) Mean and SE of the macrophage counts in the BAL of all animals. Animals that received elastase (ELA and ELABC) had greater values than SAL and SALBC groups (^*∗*^*p* < 0.05). There were no differences between the ELA and ELABC groups. (c) Mean and SE of neutrophil counts from the BAL samples of all experimental groups. The elastase-induced group (ELA) had greater values than the control groups (^*∗*^*p* < 0.05). The treatment with proteinase inhibitor (ELABC) attenuated this increase (^#^*p* < 0.05). (d) Mean and SE of the lymphocyte counts in the BAL of all animals. The elastase-induced groups (ELA and ELABC) had greater values than the control groups SAL and SALBC (^*∗*^*p* < 0.05). There were no differences between the ELA and ELABC groups.

Figure 4(a) shows the leukocyte counts in the BAL samples from all the experimental groups. The elastase-induced group had a greater total number of cells than the control groups (*p* < 0.05). In the ELABC group we observed a decrease in the total number of leukocytes compared to the elastase-induced group (*p* < 0.05).

The macrophage counts in the BAL samples from all the experimental groups are presented in Figure 4(b). The elastase-induced groups (ELA and ELABC) had greater values than the control groups (SAL and SALBC, *p* < 0.05). There were no differences between the ELA and ELABC groups.

Figure 4(c) also shows the neutrophil counts from the BAL samples from all the experimental groups. The elastase-treated group had greater values than the control groups (*p* < 0.05). The animals that received elastase and were treated with BbCI (ELABC) had decreased neutrophil counts compared to the ELA group (*p* < 0.05). The lymphocyte counts from the BAL samples from all the experimental groups are also shown in Figure 4(a). The elastase-induced groups (ELA and ELABC) had greater values than the control groups (SAL and SALBC, *p* < 0.05). There were no differences between the ELA and ELABC groups.

### 3.3. Morphometric Analysis

#### 3.3.1. Mean Linear Intercept (Lm)

**Figure 5:**
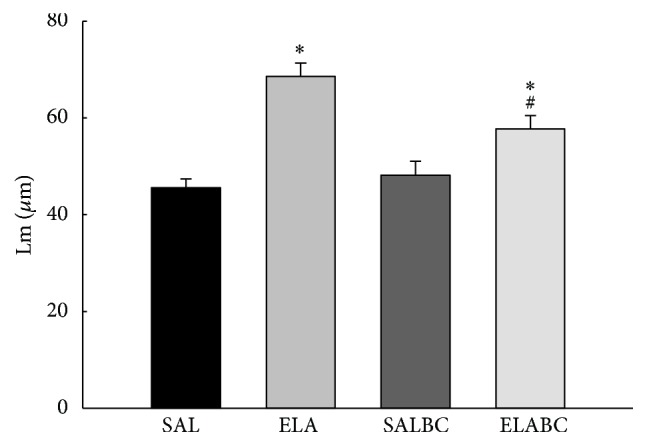
Mean and SE of Lm values and the representative photomicrographs, of the four experimental groups. Elastase-induced groups (ELA and ELABC) had greater values than control groups (^*∗*^*p* < 0.05). Animals that received elastase and were treated with BbCI (ELABC) showed a decrease when compared with elastase-induced mice (ELA) (^#^*p* < 0.05).

Figure 5 shows the Lm values for all the experimental groups. The elastase-induced groups (ELA and ELABC) had greater values than the control groups (*p* < 0.05). The animals that received elastase and were treated with BbCI (ELABC) had decreased Lm measurements compared to ELA (*p* < 0.05).

#### 3.3.2. Lung Inflammation

The inflammatory cells count present in the alveolar septa is shown in Tables 1 and 2 for all the experimental groups. In the alveolar septa, we found an increase in neutrophils, macrophages, and TNF*α*-positive cells in the ELA group compared to controls (*p* < 0.05). The treatment with the proteinase inhibitor BbCI, in the animals that received elastase, attenuated the increase of neutrophils and macrophages in the alveolar septa compared with the ELA group (*p* < 0.05). There were no differences between the SAL and SALBC groups.

When we analyzed the airways, we obtained similar results. There was an increase in neutrophils and TNF*α*-positive cells in the airway walls in ELA group compared with the SAL and SALBC groups (*p* < 0.05). Treatment with the proteinase inhibitor BbCI in the ELABC group reduced the number of neutrophils and TNF*α*-positive cells in the airway walls compared with the ELA group (*p* < 0.05). There were no differences between the SAL and SALBC groups.

#### 3.3.3. Extracellular Matrix Remodeling

The analysis of cells staining positively for extracellular matrix is shown in Table 1 for cells in the alveolar septa and in Table 2 for cells in the airway walls for all the experimental groups. In the alveolar septa, there was an increased volume fraction of collagen and elastic fibers in the ELA group compared with the SAL and SALBC groups (*p* < 0.05). In the ELABC group, there was a reduction in the volume fraction of collagen and elastic fibers compared with the ELA group (*p* < 0.05). The collagen fiber values for the ELABC group were higher than those for the SAL and SALBC groups (*p* < 0.05). When we analyzed the airway wall remodeling, we obtained similar results. There was an increase in the volume fraction of collagen and elastic fibers in the ELA group compared with the SAL and SALBC groups (*p* < 0.05). Treatment with the proteinase inhibitor in the ELABC group reduced the volume fraction of the collagen and elastic fibers compared with the ELA group (*p* < 0.05).

We observed an increase in the number of MMP-9-, MMP-12-, and TIMP-1-positive cells in the alveolar septa of the ELA group compared with the SAL and SALBC groups (*p* < 0.05). In the ELABC group the number of MMP-9-, MMP-12-, and TIMP-1-positive cells in the alveolar septa was diminished compared with the ELA group (*p* < 0.05). In the airway walls of the ELABC group, the number of MMP-9- and MMP-12-positive cells was higher than in the SAL and SALBC groups (*p* < 0.05). The airway wall analysis revealed increased numbers of MMP-9-, MMP-12-, and TIMP-1-positive cells in the ELA group compared with the SAL and SALBC groups (*p* < 0.05). In the ELABC group, the number of MMP-9- and MMP-12-positive cells was decreased in the airway walls compared with the ELA group (*p* < 0.05). However, there were no differences in the number of TIMP-1-positive cells between the groups.

#### 3.3.4. Oxidative Stress

The positively stained oxidative stress cell counts in the alveolar septa airway walls are shown in Tables 1 and 2, respectively, for all the experimental groups. In the alveolar septa, there was an increase in iNOS- and eNOS-positive cells in the ELA group compared with the SAL and SALBC groups (*p* < 0.05). In the ELABC group, we observed a reduction in the number of iNOS-positive cells compared with the ELA group (*p* < 0.05). However, the number of eNOS-positive cells was unchanged. The number of iNOS-positive cells in the ELABC group increased compared with the SAL and SALBC groups (*p* < 0.05). When we analyzed the airway walls, we observed similar results. There was an increase in iNOS- and eNOS-positive cells in the ELA group compared with the SAL and SALBC groups (*p* < 0.05). In the ELABC group, the number of iNOS-positive cells was reduced compared with the ELA group (*p* < 0.05). There was an increase in the iNOS-positive cells in the ELABC group compared with SAL and SALBC groups (*p* < 0.05).

The volume fraction of 8-iso-PGF2*α* in the alveolar septa analysis revealed an increase in the ELA groups compared with the SAL and SALBC groups (*p* < 0.05). We found that the proteinase inhibitor BbCI attenuated this increase (*p* < 0.05) compared with the ELA animals. However, in the airway walls, the volume fraction of 8-iso-PGF2*α* in the ELA and ELABC groups was increased compared with the SAL and SALBC groups (*p* < 0.05). However, there was no difference between the ELA and ELABC groups.

### 3.4. Mucin-Positive Cells

**Figure 6:**
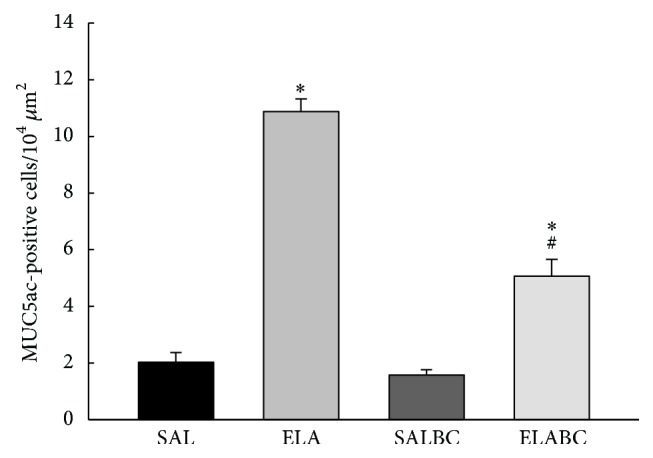
Mean and SE of positive MUC5ac cells, in airways of all experimental groups. Animals that received elastase (ELA) had greater values than SAL and SALBC groups (^*∗*^*p* < 0.05). In the ELABC group we observed a reduction in the number of MUC5ac-positive cells compared with ELA group (^#^*p* < 0.05).

The counts for MUC5ac-positive cells in the airway walls are shown in Figure 6 for all the experimental groups. There was an increase in the number of MUC5ac-positive cells in the airways of the ELA and ELABC groups compared with the SAL and SALBC groups (*p* < 0.05). In the ELABC group we observed a reduction in the number of MUC5ac-positive cells compared with the ELA group (*p* < 0.05).

### 3.5. Qualitative Analysis

**Figure 7:**
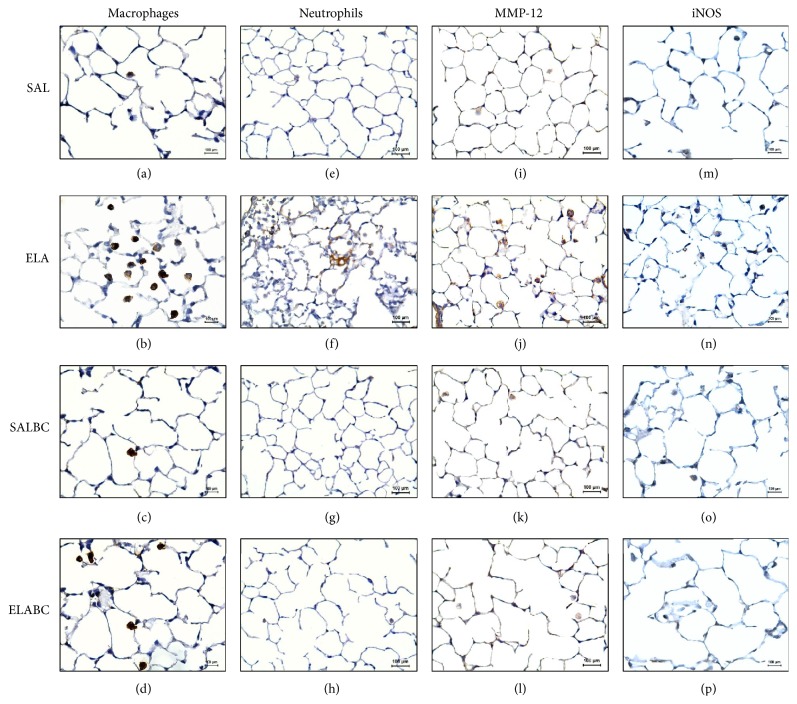
Representative photomicrographs of mice lung alveolar septum of four experimental groups submitted to immunohistochemistry: macrophages (a, b, c, and d panels, ×400), neutrophils (e, f, g, and h panels, ×400), MMP-12 (i, j, k, and l panels, ×400), and iNOS (m, n, o, and p panels, ×400).

Representative immunohistochemistry photomicrographs of the murine lung alveolar septa of the four experimental groups are shown in Figure 7. The sections were stained to identify macrophages ((a), (b), (c), and (d) panels, ×400) or neutrophils ((e), (f), (g), and (h) panels, ×400) or to stain for MMP-12 ((i), (j), (k), and (l) panels, ×400) or iNOS ((m), (n), (o), and (p) panels, ×400). There was an increase in the number of macrophages, neutrophils, and MMP-9- and iNOS-positive cells in the elastase-treated animals that did not receive the proteinase inhibitor (ELA group: (b), (f), (j), and (n), respectively) compared with the saline-treated animals (SAL group: (a), (e), (i), and (m); SALBC group: (c), (g), (k), and (o)). As shown in Figure 7, the proteinase inhibitor treatment reduced the number of macrophages (d), neutrophils (h), and MMP-9- (l) and iNOS-positive cells (p).

**Figure 8:**
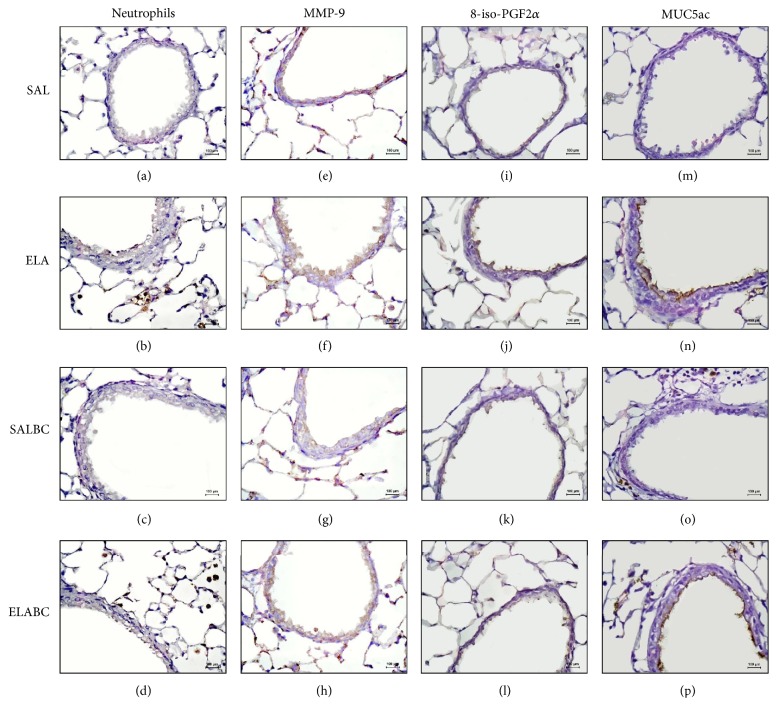
Representative photomicrographs of mice lung airways walls of all experimental groups submitted to immunohistochemistry: neutrophils (a, b, c, and d panels, ×400), MMP-9 (e, f, g, and h panels, ×400), 8-iso-PGF2*α* (i, j, k, and l panels, ×400), and MUC5ac (m, n, o, and p panels, ×400).

Figure 8 shows representative immunohistochemistry photomicrographs of the murine airway walls from all the experimental groups stained for neutrophils ((a), (b), (c), and (d) panels, ×400) or MMP-9 ((e), (f), (g), and (h) panels, ×400), 8-iso-PGF2*α* ((i), (j), (k), and (l) panels, ×400), or MUC5ac ((m), (n), (o), and (p) panels ×400). The number of neutrophils and cells positive for MMP-9, 8-iso-PGF2*α*, and MUC5ac in the animals treated with elastase was increased (ELA group: (b), (f), (j), and (n), resp.) compared with the saline-treated animals (SAL group: (a), (e), (i), and (m); SALBC group: (c), (g), (k), and (o)). As shown in Figure 8, the BbCI treatment reduced the number of neutrophils (d) and cells positive for MMP-9 (h), 8-iso-PGF2*α* (l), and MUC5ac (p).

## 4. Discussion

In the present study, we tested the hypothesis that the proteinase inhibitor BbCI from plants ameliorates elastase-induced pulmonary changes in mice. We showed that BbCI decreased the elastase-induced lung mechanics abnormalities and increases in BAL cell counts. It also reduced the inflammatory, extracellular matrix remodeling, and oxidative stress responses in the alveolar septa and airway walls of mice.

The role of proteinases in emphysema is widely accepted. However, there are uncertainties about which cells and/or proteinases have key functions in the development and progression of the disease. Publications variably report that serine, cysteine, and/or metalloproteinase classes are the most likely proteinases involved in COPD pathogenesis [16–18].

Neutrophil elastase, proteinase 3, and cathepsin G are serine class proteinases stored by polymorphonuclear neutrophils (PMN) and monocytes. They are released when proinflammatory mediators induce PMN and monocyte degranulation. These serine proteinases have been linked to lung parenchyma destruction and mucus production [19]. The cysteine class of proteinases includes cathepsin S and L which are both potent elastases. They promote macrophage-mediated extracellular matrix protein degradation. The metalloproteinase class includes MMP-1, MMP-2, MMP-9, and MMP-14 and members of the ADAM family of proteinases. In addition to promoting collagen and elastin degradation, metalloproteinases also increase MUC5ac expression by airway epithelial cells [19, 20].

Recently, the development of novel therapeutics targeting proteinases has been discussed for several diseases. Proteinases are no longer regarded as only extracellular matrix degrading enzymes. Some proteinases also have important signaling functions involved in numerous crucial biological processes. Thus, more selective proteinase inhibitors are being developed to target these processes [6, 21, 22]. Synthetic molecules have been developed to assess the therapeutic efficacy of proteinases in COPD, including CP-471474 [23], ZD0892 [24], SP-B [25], MR889 [26], and FR901277 [27].

To assess the efficacy of the BbCI treatment on elastase-induced pulmonary pathologies in mice, we evaluated lung mechanics 28 days after the elastase instillation. Our results showed that the BbCI treatment reduced the respiratory elastance (Ers) and resistance (Rrs). The decrease in respiratory resistance (Rrs) was observed in both groups that received BbCI (SALBC and ELABC) compared to SAL group. These results suggest that BbCI might have an airway bronchodilator effect that should be tested in future studies using experimental models of asthma. We have previously shown, using another proteinase inhibitors, bronchodilator effects of this class of inhibitor [28, 29].

The BbCI also reduced the tissue resistance elastance (Htis) and airway resistance (Raw). These results indicate that intratracheal elastase altered the function of both the airways and lung parenchyma. In contrast to the results in our study, previous studies showed that respiratory resistance did not change in elastase-induced models of emphysema [30, 31]. Some papers report changes in lung mechanics. Using an elastase-induced model of emphysema, Ito et al. [31] showed a decrease in respiratory system elastance. The authors suggest that abnormal collagen remodeling plays a key role in pulmonary function and changes in mechanical forces that occur during the development of emphysema [31]. Using an elastase-induced animal model, Hantos et al. [30] showed a decrease in tissue elastance and no alterations in airway or tissue resistance. In marked contrast to other studies, the authors of the latter study concluded that tissue destruction is not linked to pulmonary dysfunction [30]. However, Scuri et al. [32] showed that elastase treatment leads to increased production of bradykinin which increases respiratory resistance and elastance. The authors showed that an antagonist of the bradykinin B2 receptor blocks this response. These data suggest that the kallikrein-kinin system is involved in this process [32]. Importantly, although all these studies used an elastase model, the protocols and doses of elastase delivered to the animals were significantly different.

In our study, the Lm analysis, which evaluates airspace enlargement, showed that the proteinase inhibitor BbCI protects against elastase-induced increases in alveolar size. This effect may be due to the ability of BbCI to inhibit proteinases released during elastase-induced inflammation in the distal lung parenchyma. These results corroborate previous biochemical assays that show that BbCI inhibits both neutrophil elastase and cathepsin G, both of which have been implicated in emphysema development [33].

Guarnieri et al. [25] used a high dose elastase-induced model of emphysema and showed that a peptide-elastase inhibitor bound to trifluoromethyl prevented enlargement of the alveoli of mice that received elastase. Suzuki et al. [34] studied the effects of curcumin in an animal model of emphysema and showed that the yellow pigment from turmeric reduced the Lm that developed in mice treated with elastase or chronically exposed to cigarette smoke. Shapiro et al. [3] studied neutrophil elastase-deficient mice exposed to cigarette smoke and showed that these mice had a 59% reduction in emphysema development (measured by Lm) compared with smoke-exposed wild type mice.

The anti-inflammatory effect of BbCI was also observed in the bronchoalveolar lavage samples in our study. The total number of BAL leukocytes and neutrophils was decreased in the animals that received elastase and were treated with the proteinase inhibitor. These findings could be due to an inhibition of the neutrophil elastase-mediated recruitment of inflammatory cells to the lung [3].

We also evaluated the neutrophil, macrophages, and TNF*α*-positive cell counts in the lung tissue as well as the BAL samples. We observed that BbCI decreased the number of macrophages and neutrophils positive cells in the alveolar septa and neutrophils and TNF*α*-positive cells in the airway walls.

Taken together, these results demonstrate important anti-inflammatory activity for BbCI in this animal model. Oliveira et al. [35] showed that pretreatment with BbCI effectively reduces neutrophil migration into the pleural cavity after carrageenan-induced inflammation. The authors also showed that BbCI decreased the number of rolling and adherent leukocytes, as well as leukocyte migrations into the spermatic fascia microcirculation after inducing an inflammatory response [35]. In a model of pulmonary edema caused by activation of neutrophils, the proteinase inhibitor BbCI decreased the edema to the same degree as a substance used as a reference. The authors concluded that BbCI is a useful tool for studying the role of neutrophil elastase in pathophysiological processes [12].

We also analyzed the effect of BbCI on elastase-induced extracellular matrix remodeling by measuring the volume fraction of collagen and elastic fibers and counting MMP-9-, MMP-12-, and TIMP-1-positive cells in the alveolar septa and airway walls. We observed that the BbCI treatment reduced the volume fraction of elastic and collagen fibers in alveolar septa and airway walls. Comparing two models of emphysema in mice, Lopes et al. [36] showed that elastase-induced mice had higher levels of lung elastin than animals exposed to smoke for 6 months. Collagen type I had the same pattern in the two groups. The patterns were not significantly different. However, when they measured lung levels of type III collagen after 6 months of smoke exposure, these animals had higher values than the elastase-treated mice [36]. We observed that the proteinase inhibitor BbCI decreased the number of MMP-9- and MMP-12-positive cells in both the alveolar septa and airway walls. Hanaoka et al. [37] studied the effects of carbocysteine in cigarette smoke-exposed rats. They reported that MMP-9 expression increased in lung tissue when animals were exposed to smoke, and a carbocysteine treatment reduced this increase [37]. Hautamaki et al. [38] studied MMP-12 deficient mice chronically exposed to cigarette smoke and demonstrated that MMP-12 is essential for the development of cigarette smoke-induced emphysema [38]. Although high levels of MMP-9 increased TIMP-1 levels to restore homeostasis, we did not find that BbCI changed the TIMP-1 levels in the airways of the elastase-treated lung.

Regarding the oxidative stress response, our data showed that the proteinase inhibitor BbCI decreased the number of iNOS-positive cells in both the alveolar septa and airway walls. It also reduced the volume fraction of 8-iso-PGF2*α* in the alveolar septum. BbCI did not reduce the number of eNOS-positive cells in either site or isoprostane levels in airways walls. Prado et al. [39] observed that iNOS inhibition attenuated the airway constriction response in animals with chronic allergic inflammation [39]. Taken together, iNOS plays a key role in modulating airway tone and likely explains the airway resistance alterations in our model.

Because mucus hypersecretion is an important pathology in COPD and proteinases, including neutrophil elastase [40], play a key role in this process, we evaluated the number of MUC5ac-positive cells in the airway walls in our experimental groups of mice. Our results showed that BbCI reduced the number of MUC5ac-positive cells in the airways of mice likely by inhibiting neutrophil elastase.

## 5. Conclusion

In conclusion, BbCI was shown to be effective in reducing elastase-induced pulmonary mechanics changes, lung tissue destruction, inflammatory alterations, and extracellular matrix remodeling and oxidative stress in animals that have received intratracheal elastase. Nevertheless, further studies are needed to elucidate the mechanisms by which BbCI protects the lung. Based on our findings, it may be considered as a potential therapeutic tool in the COPD management.

## Figures and Tables

**Table 1:** Effect of BbCI treatment on positive cells in alveolar septum of all groups. Data are presented as mean ± SE. ELA group had greater values than control groups (SAL and SALBC) in all alveolar septa parameters. The group that was treated with BbCI inhibitor (ELABC) had attenuated this response in macrophages, neutrophils, collagen fibers, elastic fibers, MMP-9- and MMP-12-positive cells, and iNOS- and 8-iso-PGF2*α*-positive cells. ^*∗*^*p* < 0.05 compared to SAL and SALBC groups. ^#^*p* < 0.05 compared to ELA group.

Alveolar septum				
Inflammatory response	SAL	ELA	SALBC	ELABC

Macrophages (cells/10^4^ *μ*m^2^)	3.55 ± 0.3	17.50 ± 2.3^*∗*^	5.00 ± 0.3	9.48 ± 1.1^*∗*,#^
Neutrophils (cells/10^4^ *μ*m^2^)	5.87 ± 0.6	20.03 ± 0.7^*∗*^	9.33 ± 0.7	13.01 ± 0.4^#^
TNF*α* (cells/10^4^ *μ*m^2^)	2.21 ± 0.2	10.41 ± 0.6^*∗*^	2.43 ± 0.3	10.01 ± 0.4^*∗*^

Remodelling response	SAL	ELA	SALBC	ELABC

Collagen fibers (%)	8.80 ± 0.1	12.12 ± 0.1^*∗*^	8.96 ± 0.1	9.98 ± 0.2^*∗*,#^
Elastic fibers (%)	0.32 ± 0.03	0.43 ± 0.03^*∗*^	0.37 ± 0.02	0.34 ± 0.02^#^
MMP-9-positive cells/10^4^ *μ*m^2^	8.63 ± 1.2	20.69 ± 1.3^*∗*^	9.89 ± 0.7	11.92 ± 0.1^#^
MMP-12-positive cells/10^4^ *μ*m^2^	10.88 ± 1.4	26.17 ± 2.5^*∗*^	11.22 ± 0.5	12.20 ± 0.6^#^
TIMP-1-positive cells/10^4^ *μ*m^2^	3.55 ± 0.3	17.50 ± 2.3^*∗*^	5.00 ± 0.3	9.48 ± 1.1^#^

Oxidative stress response	SAL	ELA	SALBC	ELABC

iNOS-positive cells/10^4^ *μ*m^2^	5.51 ± 0.2	20.55 ± 1.2^*∗*^	6.03 ± 0.5	12.97 ± 1.1^*∗*,#^
eNOS-positive cells/10^4^ *μ*m^2^	3.06 ± 0.2	8.93 ± 0.4^*∗*^	3.58 ± 0.2	8.13 ± 0.4
8-iso-PGF2*α*-positive cells/10^4^ *μ*m^2^	6.17 ± 0.6	16.52 ± 0.6^*∗*^	6,74 ± 0.3	11.07 ± 1.1^#^

**Table 2:** Effect of BbCI treatment on positive cells in airway walls of all groups. Data are presented as mean ± SE. ELA group had greater values than control groups (SAL and SALBC) in all airway walls parameters. The group that was treated with BbCI inhibitor (ELABC) had attenuated this response in neutrophils, TNF*α*, collagen fibers, elastic fibers, MMP-9- and MMP-12-positive cells, and iNOS-positive cells. ^*∗*^*p* < 0.05 compared to SAL and SALBC groups. ^#^*p* < 0.05 compared to ELA group.

Airway walls				
Inflammatory response	SAL	ELA	SALBC	ELABC

Neutrophils (cells/10^4^ *μ*m^2^)	2.77 ± 0.3	11.36 ± 0.8^*∗*^	5.03 ± 0.4	6.23 ± 0.3^#^
TNF*α* (cells/10^4^ *μ*m^2^)	2.58 ± 0.4	10.62 ± 0.8^*∗*^	2.92 ± 0.3	5.27 ± 0.6^#^

Remodelling response	SAL	ELA	SALBC	ELABC

Collagen fibers (%)	5.55 ± 0.1	8.83 ± 0.1^*∗*^	5.61 ± 0.1	6.02 ± 0.2^#^
Elastic fibers (%)	0.81 ± 0.1	2.23 ± 0.5^*∗*^	1.08 ± 0.0	1.12 ± 0.4^#^
MMP-9-positive cells/10^4^ *μ*m^2^	2.07 ± 0.4	12.82 ± 0.6^*∗*^	4.63 ± 0.8	6.59 ± 0.7^*∗*,#^
MMP-12-positive cells/10^4^ *μ*m^2^	3.14 ± 0.3	13.69 ± 0.9^*∗*^	4.13 ± 0.2	8.50 ± 0.7^*∗*,#^
TIMP-1-positive cells/10^4^ *μ*m^2^	2.96 ± 0.2	8.69 ± 0.8^*∗*^	2.85 ± 0.6	8.08 ± 0.6^*∗*^

Oxidative stress response	SAL	ELA	SALBC	ELABC

iNOS-positive cells/10^4^ *μ*m^2^	5.35 ± 0.5	20.42 ± 1.1^*∗*^	6.24 ± 0.7	12.78 ± 1.2^*∗*,#^
eNOS-positive cells/10^4^ *μ*m^2^	3.20 ± 0.2	8.86 ± 0.7^*∗*^	3.80 ± 0.3	7.96 ± 0.4
8-iso-PGF2*α*-positive cells/10^4^ *μ*m^2^	5.91 ± 0.8	15.36 ± 1.9^*∗*^	7.65 ± 0.5	15.30 ± 1.2^*∗*^
